# Ibrutinib Modulates Proliferation, Migration, Mitochondrial Homeostasis, and Apoptosis in Melanoma Cells

**DOI:** 10.3390/biomedicines12051012

**Published:** 2024-05-04

**Authors:** Fernanda Vitelli Lins, Elizabete Cristina Iseke Bispo, Naomí Souza Rodrigues, Maria Victória Souto Silva, Juliana Lott Carvalho, Guilherme Martins Gelfuso, Felipe Saldanha-Araujo

**Affiliations:** 1 Laboratório de Hematologia e Células-Tronco, Faculdade de Ciências da Saúde, Universidade de Brasília, Brasília 70910-900, DF, Brazil; fernanda.lins@utsouthwestern.edu (F.V.L.); elizabete.iseke@hotmail.com (E.C.I.B.); naomi.sr04@gmail.com (N.S.R.); 2 Children’s Medical Center Research Institute, University of Texas Southwestern Medical Center, Dallas, TX 75390, USA; 3 Laboratório Interdisciplinar de Biociências, Faculdade de Medicina, Universidade de Brasília, Brasília 70910-900, DF, Brazil; vicsoutosilva@gmail.com (M.V.S.S.); julianalott@gmail.com (J.L.C.); 4 Laboratório de Medicamentos, Alimentos e Cosméticos, Universidade de Brasília, Brasília 70910-900, DF, Brazil; gmgelfuso@unb.br

**Keywords:** BTK, Ibrutinib, melanoma, MeWo, skin cancer

## Abstract

Ibrutinib, a tyrosine kinase inhibitor with a broad spectrum of action, has been successfully explored to treat hematological and solid cancers. Herein, we investigated the anti-cancer effect of Ibrutinib on melanoma cell lines. Cytotoxicity was evaluated using the MTT assay. Apoptosis, mitochondrial membrane potential, reactive oxygen species (ROS) production, cell proliferation, and cell cycle stages were determined by flow cytometry. LDH release and Caspase 3/7 activity were determined by colorimetric and luminescent assays, respectively. Cell migration was evaluated by wound scratch assay. Gene expression was determined by real-time PCR. Gene Ontology (GO) enrichment analysis of melanoma clinical samples was performed using the Database for Annotation, Visualization and Integrated Discovery (DAVID). MTT assays showed that Ibrutinib is toxic for MeWo, SK-MEL-28, and WM164 cells. The annexin V/PI staining, Caspase 3/7 activity, and LDH release in MeWo cells revealed that apoptosis is the primary mechanism of death caused by Ibrutinib. Corroborating such observation, we identified that Ibrutinib treatment impairs the mitochondrial membrane potential of such cells and significantly increases the transcriptional levels of the pro-apoptotic factors *ATM*, *HRK*, *BAX*, *BAK*, *CASP3*, and *CASP8*. Furthermore, Ibrutinib showed antimetastatic potential by inhibiting the migration of MeWo cells. Finally, we performed a functional enrichment analysis and identified that the differential expression of Ibrutinib-target molecules is associated with enrichment of apoptosis and necrosis pathways in melanoma samples. Taken together, our results clearly suggest that Ibrutinib can be successfully explored as an effective therapeutic approach for melanomas.

## 1. Introduction

Melanoma is a malignant tumor originated from melanocytes. As their main function, these cells have the synthesis of melanin by melanosomes, which are then transferred to keratinocytes by dendritic pathways [1,2,3,4]. Melanin plays various biological roles, such as hair and skin pigmentation, and, as a UV absorbent and antioxidant molecule, it also protects cells from UV-induced damage and oxidative stress [5,6].

Melanoma accounts for 1% of all types of skin cancer, but it is responsible for more than 80% of deaths related to this disease group, representing a high burden for healthcare worldwide [6]. In Brazil, it is responsible for about 3% of all skin cancers; however, it is the most serious due to the high probability of causing metastasis. According to the Brazilian National Institute of Cancer, the country registered a total of 1923 deaths from melanoma in 2020. In the US, the incidence of melanoma has been increasing to about 3% every year, and the Centers for Disease Control and Prevention recorded a total of 8092 deaths from melanoma in 2019. In Europe, the melanoma incidence is even more concerning, with countries like Norway and Denmark presenting the highest incidence of 29.6 and 27.6 new cases per 100,000 persons, respectively. Australia and New Zealand have the highest incidence among all countries, with 33.6 and 33.3 new cases per 100,000 people, respectively [7,8].

The molecular landscape of melanoma comprises driver mutations in genes that are related to cell proliferation (*BRAF*, *NRAS*, and *NF1*), growth and metabolism (*PTEN* and *KIT*), apoptosis resistance (*P53*), cellular replication (*TERT*), cellular identity (*ARID2*), and cell cycle regulation (*CDKN2A*) [9]. Mutations in *BRAF* can be found in about 50% of melanomas and are mainly associated with cell proliferation. Nevertheless, mutations in *BRAF* have also been correlated with tumor invasion, cell migration, metastasis, and immune escape. *NRAS* also stands out as a relevant driver of melanoma since its mutations are detected in approximately 20% of all cases. Clinically, those are related to aggressive tumors and a higher rate of lymph node metastasis [6,10].

Currently, the primary treatment strategy for cutaneous melanoma diagnosed at early stages is local resection. However, when melanoma is diagnosed at later stages and has already aggravated and generated metastases, surgery is unlikely to be effective due to the low accessibility to the tumor and the uncertain number of metastatic lesions [11]. In this scenario, chemotherapy has been a notable advancement in melanoma treatment. However, the adverse effects and tumor resistance constitute important limiting factors for this therapeutic strategy [12]. Recently, adoptive cell therapy treatment approaches, such as the use of chimeric antigen receptor T cells (CAR-T), have been explored for treating melanoma. Despite the increased interest in CAR-T cells, the difficulty of promoting drug penetration and toxicity are still significant challenges that must be overcome to achieve effective clinical application [2].

Ibrutinib is a Bruton’s tyrosine kinase (BTK) inhibitor best known for its effectiveness in treating certain B-cell malignancies. Its target is the Bruton’s Tyrosine Kinase (BTK), which binds to the amino acid Cys 481 located in the ATP domain [13,14,15]. Given that the cysteine residue targeted by Ibrutinib is also present in other tyrosine kinase proteins, Ibrutinib is able to act on additional molecular targets, such as those from the TFK family members (ITK, TEC, BMX, and RLK), EGFR family members (EGFR, ERBB2/HER2, and ERBB4/HER4), and two other kinases BLK and JAK 3 [16]. Consequently, Ibrutinib is one of the few tyrosine kinase inhibitors being explored as a treatment strategy in both solid and non-solid tumors. Melanoma, while distinct in origin and characteristics, shares some commonalities with other cancers regarding signaling pathways and molecular mechanisms. In this sense, the rationale for considering Ibrutinib in melanoma treatment might involve its ability to disrupt critical signaling pathways, potentially inhibiting the growth and survival of melanoma cells. However, the effect of Ibrutinib on melanoma is still not completely elucidated.

Recognizing the significance of several Ibrutinib targets in melanoma, we hypothesized that this drug may be toxic to metastatic melanoma cell lines. Therefore, in this study, we propose investigating the anti-cancer effect of Ibrutinib on melanoma cell lines, identifying the cellular and molecular changes promoted by the drug. Furthermore, we performed an in silico analysis accessing a cohort of 144 patients with melanoma to evaluate which biological processes are modulated by Ibrutinib target molecules in this kind of cancer. The aim is to pave the way for the use of Ibrutinib for an effective melanoma treatment.

## 2. Material and Methods

### 2.1. Cell Lines and Cell Culture

MeWo, SK-MEL-28, and WM164 cell lines were used as models of metastatic melanoma in this study. MeWo and SK-MEL-28 were purchased from ATCC, and the WM164 cell line was kindly provided by Prof. Silvya Stuchi Maria-Engler (University of São Paulo, São Paulo, Brazil). As a control, human foreskin fibroblasts (HFF-1, ATCC) were used. Cells were expanded using RPMI media supplemented with 10% FBS, 2 mmol/L penicillin/streptomycin, 2 mmol/L glutamine, and 10 mmol/L HEPES (all reagents were acquired from Sigma-Aldrich, St. Louis, MO, USA). After expansion, the cells were harvested using trypsin EDTA (0.5%) and stored in liquid nitrogen until the following experiments were carried out. Ibrutinib (Cayman Chemical, Ann Arbor, MI, USA) was dissolved in dimethylsulfoxide (DMSO) (Sigma-Aldrich, St Louis, MO, USA) at a concentration of 20 mmol/L and stored at −20 °C until use.

### 2.2. MTT Assay

The effect of Ibrutinib (Cayman Chemical, Ann Arbor, MI, USA) on the melanoma cell lines was evaluated by the MTT assay. Briefly, 7 × 10^3^ cells were seeded in 96-well plates. After 24 h, they were treated with different concentrations of Ibrutinib (1, 2, 2.5, 3, 5, 10, 25, 50 and 100 μmol/L) for 48 h. Following, 10 μL of MTT reagent (5 mg/mL; Sigma-Aldrich, St. Louis, MO, USA) was added to each well and the plates were incubated for 4 h protected from light. After this period, the MTT solution was removed, replaced by 100 μL of dimethylsulfoxide (DMSO), and the plate was homogenized for 15 min to dissolve the formed formazan crystals. Absorbance was measured in the spectrophotometer Synergy H1 (Agilent BioTek, Santa Clara, CA, USA) at 570 nm. Then, the IC_50_ dose of Ibrutinib was determined.

### 2.3. Clonogenic Assay

MeWo cell line was plated onto 6-well plates at a density of 1000 cells and treated with Ibrutinib at IC_25_ and IC_50_ doses. After ten days of culture, the colonies were fixed with 4% paraformaldehyde and stained with a 1% crystal violet solution. Then, the number of colonies was quantified using ImageJ software (National Institutes of Health, Bethesda, MD, USA, version 1.53o). Cell groups containing at least 30 cells were considered to be colonies.

### 2.4. Apoptosis Assay

Cell apoptosis was detected using annexin-V and Propidium Iodide (PI) staining (BD Biosciences, East Rutherford, NJ, USA; and Invitrogen, Waltham, MA, USA, respectively). Using 12 well plates, 8.5 × 10^4^ cells were seeded. After 24 h, cells were treated with Ibrutinb at the IC_50_ dose for 48 h. Then, cells were trypsinized, washed with PBS, and stained with annexin-V diluted in the binding buffer. Finally, the cells were stained with PI and analyzed using flow cytometry (FACS Calibur, BD Biosciences, East Rutherford, NJ, USA). A total of 10,000 events were recorded for each sample, and the data were analyzed using FlowJo software 10.0.7 (Treestar Inc., Ashland, OR, USA).

### 2.5. Lactate Dehydrogenase (LDH) Release

LDH release in supernatants of cells (8.5 × 10^4^) treated with the IC_50_ dose of Ibrutinib was determined using CytoTox 96^®^ Non-Radioactive Cytotoxicity Assay, according to the manufacturer’s instructions (Promega Corporation, Madison, WI, USA). In brief, following a 48 h treatment with Ibrutinib, 50 μL of the CitoTox 96 reagent was added to 50 μL of cell supernatant and the plate was incubated for 30 min. Then, 50 μL of the stop solution was added to each well and the absorbance was determined at 490 nm using a DTX 800 Series Multimode Detector (Beckman Coulter, Brea, CA, USA).

### 2.6. Mitochondrial Membrane Potential

The mitochondrial membrane potential was determined by flow cytometry, using Rhodamine-123 dye (Invitrogen, Waltham, MA, USA). For this, 8.5 × 10^3^ cells were plated and, after 24 h, treated with IC_50_ dose of Ibrutinib. After 48 h of treatment, cells were dissociated with accutase and stained with Rhodamine-123 (5 μg/mL) for 30 min at room temperature. Then, cells were washed twice with PBS and analyzed by flow cytometry (FACS Calibur, BD Biosciences, East Rutherford, NJ, USA). A total of 10,000 events were recorded for each sample, and the data were analyzed using FlowJo software 10.0.7 (Treestar, Inc., Ashland, OR, USA).

### 2.7. Caspase 3/7 Activity

The Caspase 3/7 activity was determined using the Caspase-Glo 3/7 assay (Promega Corp., Madison, WI, USA). For this, 7 × 10^3^ cells were treated with the IC_50_ dose of Ibrutinib for 48 h at 37 °C. After this period, 100 μL of the reagent Caspase-Glo 3/7 was added to each well and, after homogenization, samples were incubated for 3 h at room temperature. Then, caspase 3/7 activity was determined by luminescence (Multimode Plate Reader, PerkinElmer, Waltham, MA, USA).

### 2.8. Cell Proliferation

Cell proliferation was evaluated by flow cytometry, using the CFSE (carboxyfluorescein succinimidyl ester, Sigma-Aldrich, St. Louis, MO, USA), according to the manufacturer’s instructions. Briefly, after being stained with 10 μmol/L CFSE for 10 min, cells were washed with media to remove the CFSE dye not incorporated. Then, 4 × 10^5^ cells were treated with the IC_50_ dose of Ibrutinib for 48 h. After this period, the percentage of CFSE^+^ cells was determined by flow cytometry (FACSCalibur, BD Biosciences, East Rutherford, NJ, USA). A total of 10,000 events were recorded for each sample, and the data were analyzed using FlowJo software 10.0.7 (Treestar, Inc., Ashland, OR, USA).

### 2.9. Cell Cycle

For cell cycle analysis, 4 × 10^5^ cells were seeded in 6-well plates and treated with the IC_50_ dose of Ibrutinib for 48 h. Afterwards, the cells were harvested, fixed with 70% ethanol, and stored at 4 °C for at least 2 h before analysis. For the flow cytometric analysis, the fixed cells were treated with 100 µg/mL RNAse A for 10 min and then stained with 50 µg/mL PI for 20 min. One hundred thousand events were collected using a FACSCalibur flow cytometer (BD Biosciences, East Rutherford, NJ, USA), and the data were analyzed using FlowJo software 10.0.7 (Treestar, Inc., Ashland, OR, USA).

### 2.10. Cell Migration

Cells were seeded in 6-well plates and cultured until reaching 90% confluence. After, cells were wounded using a sterile pipette tip and serum deprivation conditions were imposed. Then, cells were treated with the IC_50_ dose of Ibrutinib and the wounded area was photographed at 0, 3, and 5 days using a Motic AE2000 Inverted Microscope (×100 magnification). The area of the scratch was measured using the ImageJ software (National Institutes of Health, Bethesda, MD, USA, version 1.53o), and the analysis was performed by comparing the initial area opened by the scratch at the initial time point with the area still open at each timepoint.

### 2.11. RNA Extraction and Real Time PCR

Total RNA was obtained from cells treated with the IC_50_ dose of Ibrutinib for 48 h. To do so, the TRI Reagent (Sigma Aldrich, St. Louis, MO, USA) was used, and the resulting RNA samples were quantified using a Nanodrop One (Thermo Scientific, San Jose, CA, USA). The complementary DNA (cDNA) was synthesized from 1.5 μg of RNA using the High-Capacity Reverse Transcription Kit, following manufacturer’s instructions (Thermo Fisher. Waltham, MA, USA). The target genes evaluated were *ATM*, *BAK*, *BAX*, *BTK*, *CASP3*, *CASP8*, *EGFR*, *ERBB2*, *HRK*, *ITK*, and *JAK3.* Primer sequences are presented in Supplementary Table S1.

All reactions were performed in duplicates, and the relative fold change value was obtained by the 2^–ΔΔCt^ method [17]. To normalize sample loading, the differences in threshold cycles (ΔCt) were obtained by subtracting the Ct value for the endogenous reference (*GAPDH*) from the Ct values of the evaluated genes. The median Ct values of the samples from untreated cells (control) were used as a reference.

### 2.12. Bioinformatics Analysis

Using cBioPortal platform (https://www.cbioportal.org, accessed on 4 November 2023), we performed an in silico analysis on a public database involving 144 metastatic melanoma samples. Such samples were obtained by excision or biopsy from 144 cases (41.7% female and 58.3% male) of primary melanoma classified as advanced melanoma [18]. The identification of differentially expressed genes (DEGs) was executed after dichotomizing the Ibrutinib target genes, according to their median expression. Such analysis involved RNA sequencing data of the 144 samples. The target genes evaluated were *BTK*, *EGFR*, *ERBB2*, *JAK3*, *ITK*, and *TEC.*

The generated DEGs list was used as input in the DAVID platform (Database for Annotation, Visualization and Integrated Discovery) [19] to identify enriched signaling pathways modulated according to the median expression of the Ibrutinib target genes.

### 2.13. Statistical Analysis

At least three independent replicates for each experimental condition were performed in all assays. Unpaired Student’s *t*-test was used when comparing two groups, and ANOVA was used in comparing three groups. All results were analyzed using the GraphPad Prism 9 software (GraphPad Software Inc., La Jolla, CA, USA). The value of *p* ≤ 0.05.was considered statistically significant, and significance levels were defined as * *p* < 0.05; ** *p* < 0.01; *** *p* < 0.001; and **** *p* < 0.0001.

## 3. Results

### 3.1. Ibrutinib Impairs MeWo Cell Line Viability

**Figure 1:**
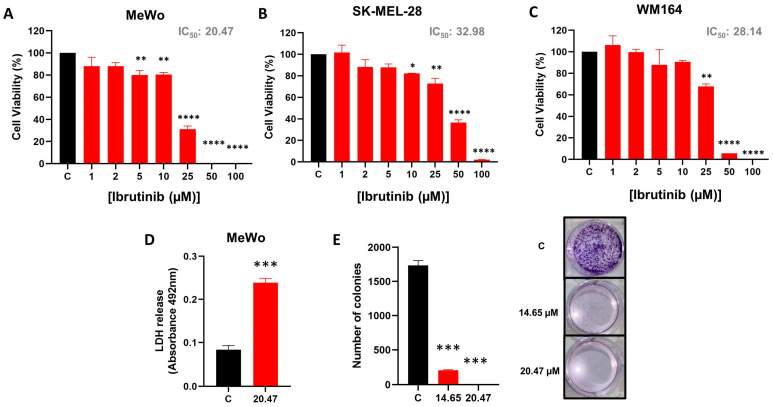
Ibrutinib induces melanoma cell death. Dose–response cytotoxicity of MeWo (**A**), SK-MEL-28 (**B**), and WM164 cells (**C**) treated with increased doses of Ibrutinib (1–100 µM). (**D**) LDH release by MeWo cells untreated (C, Control) and treated with IC_50_ dose of Ibrutinib (20.47 µM). (**E**) Clonogenic assay of the MeWo cells untreated (C, Control) and treated with IC25 (14.65 µM) or IC50 (20.47 µM) dose of Ibrutinib. Data are expressed as mean with SEM and analyzed by unpaired Student’s *t*-test or ANOVA. * *p* < 0.05; ** *p* < 0.01; *** *p* < 0.001; and **** *p* < 0.0001.

According to the MTT assay, Ibrutinib treatment significantly reduced the viability of MeWo, WM164, and SK-MEL-28 melanoma cell lines in a dose-dependent manner (Figure 1A–C). Using linear regression, we identified the IC_50_ dose of Ibrutinib for the evaluated cell lines (MeWo: 20.47 µM, WM164: 28.14 µM, and SK-MEL-28: 32.98 µM). Considering that the MeWo lineage was the most sensitive to treatment with Ibrutinib, we selected this lineage to characterize the cell death promoted by this TKI. In agreement with the observed reduction in cell viability, the IC_50_ dose of Ibrutinib induced LDH release by MeWo cells (*p* = 0.0002) (Figure 1D). Furthermore, the IC_25_ and IC_50_ dose of Ibrutinib promoted an inhibition in the number of colonies in MeWo cells (*p* = 0.0002 and *p* = 0.0001, respectively) (Figure 1E).

### 3.2. Ibrutinib Treatment Increases Annexin-V^+^ Mewo Cells

**Figure 2:**
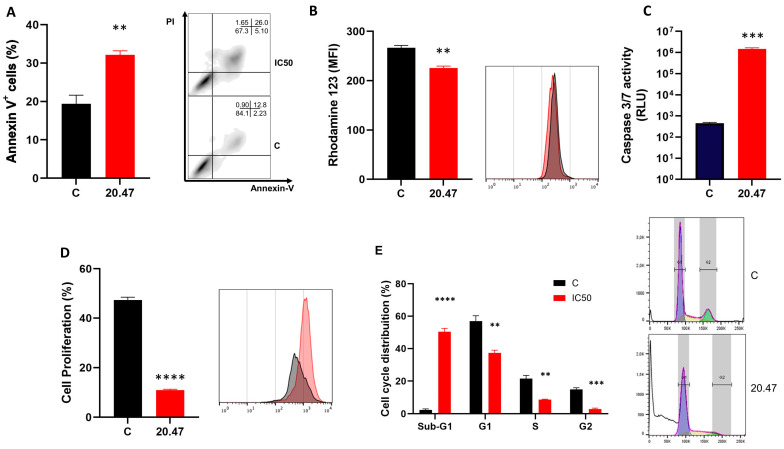
Effect of Ibrutinib on proliferation, mitochondrial membrane potential and caspase 3/7 activation in MeWo cells. (**A**) Apoptosis detection by annexin V/PI staining method in MeWo cells untreated (C, Control) and treated with IC_50_ dose of Ibrutinib (20.47 µM). Representative dot plot showing the expressions of annexin-V and PI in MeWo cells untreated (C, Control) and treated with IC_50_ dose of Ibrutinib (20.47 µM). (**B**) Mitochondrial membrane potential determined by Rhodamine 123 staining in MeWo cells untreated (C, Control) and treated with IC_50_ dose of Ibrutinib (20.47 µM). A representative histogram of MeWo cells untreated (C, Control) and treated with IC_50_ dose of Ibrutinib (20.47 µM) is presented. (**C**) Caspase 3/7 activity of MeWo cells untreated (C, Control) and treated with IC_50_ dose of Ibrutinib (20.47 µM). (**D**) Cell proliferation determined by CFSE staining of MeWo cells untreated (C, Control) and treated with IC_50_ dose of Ibrutinib (20.47 µM). A representative histogram of MeWo cells untreated (C, Control) and treated with IC_50_ dose of Ibrutinib (20.47 µM) is presented. (**E**) Phases of the cell cycle in MeWo cells untreated (C, Control) and treated with IC_50_ dose of Ibrutinib (20.47 µM). A representative histogram of MeWo cells untreated (C, Control) and treated with IC_50_ dose of Ibrutinib (20.47 µM) is presented. For flow cytometry analyzes, ten thousand events were recorded for each sample. Data are expressed as mean with SEM and analyzed by unpaired Student’s *t*-test. ** *p* < 0.01; *** *p* < 0.001; and **** *p* < 0.0001.

The increase in annexin-V-positive cells observed after treatment of the MeWo cell line with the IC_50_ dose of Ibrutinib (*p* = 0.003) indicates that apoptosis is a possible death mechanism involved in the observed cell death (Figure 2A). This finding was corroborated by annexin-V and PI staining in SK-MEL-28 cells, which also presented a significant increase in annexin-V staining when treated with the respective IC_50_ dose of Ibrutinib (Supplementary Figure S1).

### 3.3. Ibrutinib Changes the Mitochondrial Membrane Potential of Melanoma Cells

MeWo cells treated with Ibrutinib presented a significant decrease in the mitochondrial membrane potential (*p* = 0.001) after their treatment with the IC_50_ dose of Ibrutinib (Figure 2B), as assessed by Rhodamine staining.

### 3.4. Ibrutinib Induces Activation of Caspases 3/7 in Melanoma Cells

To better characterize the cell death event observed, we evaluated whether Ibrutinib could modulate the activation of caspase 3/7 in the MeWo lineage. Thus, MeWo cells treated with Ibrutinib at the IC_50_ dose showed increased activity of caspase 3/7 compared to the control group (*p* = 0.0006), which consisted of untreated cells (Figure 2C).

### 3.5. Ibrutinib Inhibits Cell Proliferation of Melanoma Cells

Importantly, in addition to compromising the viability of the MeWo cells, Ibrutinib also effectively controlled cell division in this cell line. After Ibrutinib treatment with the IC_50_ dose (*p* < 0.0001), MeWo cells presented a significant inhibition in their proliferative capacity, as assessed by CFSE staining (Figure 2D).

### 3.6. Ibrutinib Induces Cycle Arrest at the Sub-G1 Phase in Melanoma Cells

We investigated the effect of Ibrutinib on cell cycle distribution in the MeWo cell line by flow cytometry. Our results showed that the IC_50_ dose of Ibrutinib promoted a significant accumulation of MeWo cells in the sub-G1 phase (*p* < 0.0001) with a concomitant decrease in the percentages of cells in the G1 (*p* = 0.002), S (*p* = 0.001) and G2/M phases (*p* = 0.0002) (Figure 2E). This cell accumulation in the sub-G1 phase agrees with the cellular apoptosis observed previously.

### 3.7. Ibrutinib Inhibits the Migration of Melanoma Cells

**Figure 3:**
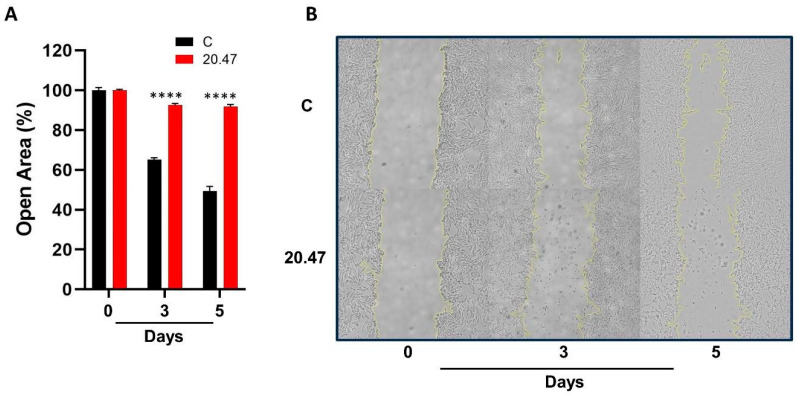
Cell migration. (**A**) Percentage of open area in the wound of MeWo cells untreated (C, Control) and treated with IC_50_ dose of Ibrutinib (20.47 µM) for 0, 3, and 5 days. (**B**) Representative wound scratch assay images of MeWo cells untreated (C, Control) and treated with IC_50_ dose of Ibrutinib for 0, 3, and 5 days. Cell migration was monitored under the microscope and quantified using the ImageJ software. Results are presented as mean ± SEM. **** *p* < 0.0001.

Cell migration is a key process in metastasis. Importantly, the IC_50_ dose of Ibrutinib promoted a significant inhibition in the migration of MeWo cells on days 3 and 5 of cell culture (*p* < 0.0001) (Figure 3A,B).

### 3.8. Real Time PCR

**Figure 4:**
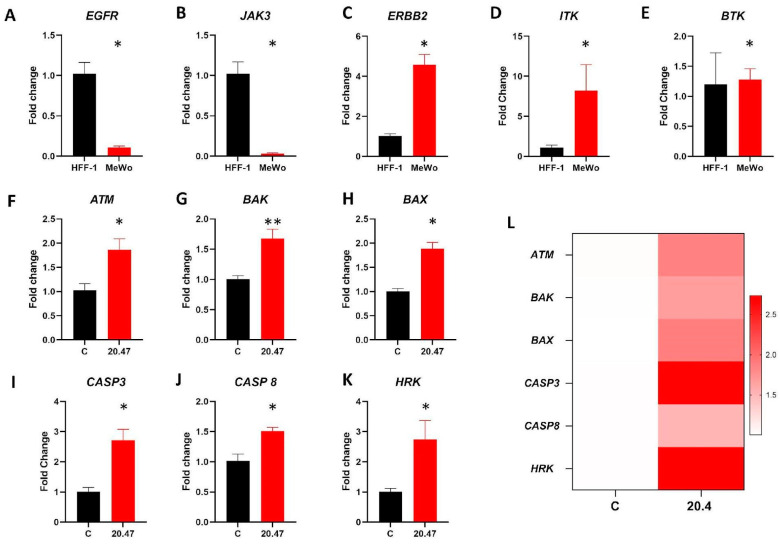
Real-time PCR. (**A**–**E**) Expression of Ibrutinib target genes *EGFR*, *JAK3*, *ERBB2*, *ITK*, and *BTK* in HFF-1 and MeWo cells. Median Ct values obtained from HFF-1 were used as a reference. (**F**–**K**) Expression of the apoptosis-related genes *ATM*, *BAK*, *BAX*, *CASP3*, *CASP8*, and *HRK* in MeWo cells untreated (C, Control) and treated with IC_50_ dose of Ibrutinib (20.47 µM). Median Ct values obtained from untreated MeWo cells (C, Control) were used as a reference. (**L**) Heatmap summarizing the expression of apoptosis-related genes in MeWo cells untreated (C, Control) and treated with IC_50_ dose of Ibrutinib (20.47 µM). All real-time PCRs reactions were performed in technical duplicate, and the relative fold change was obtained with the 2^−ΔΔCt^ method. Data are expressed as mean with SEM and analyzed by unpaired student’s *t*-test. * *p* < 0.05; ** *p* < 0.01.

Initially, we evaluated the expression of Ibrutinib target genes in MeWo and human fibroblast (HFF-1). While HFF-1 showed higher levels of *EGFR* (*p* = 0.001) and *JAK-3* (*p* = 0.001) genes compared to MeWo, the melanoma cell line presented higher expression of *ERBB2* (*p* = 0.001), and *ITK* (*p* = 0.04) (Figure 4A–D). MeWo and HFF-1 had no difference regarding the transcriptional levels of the *BTK* gene (Figure 4E).

We also analyzed genes related to apoptosis and found that, by the experiments above, MeWo cells treated with Ibrutinib presented increased levels of the proapoptotic genes *ATM* (*p* = 0.01), *BAK* (*p* = 0.007), *BAX* (*p* = 0.001), *CASP3* (*p* = 0.005), *CASP8* (*p* = 0.009), and *HRK* (*p* = 0.02) (Figure 4F–L).

### 3.9. Identification of Differentially Expressed Genes and Pathways Modulated by Ibrutinib in Clinical Samples of Melanoma

**Figure 5:**
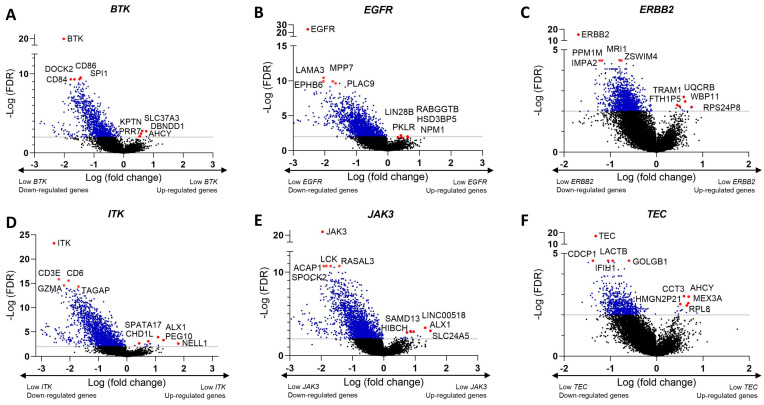
Summary of the DEGs according to the expression level of Ibrutinib target genes. (**A**–**F**) Volcano plots illustrating the down- and up-regulated genes in melanoma samples according to expression of *BTK*, *EGFR*, *ERBB2*, *ITK*, *JAK3*, and *TEC*. Genes with adjusted *p*-value < 0.05 are highlighted using blue dots.

Through the cBioPortal platform, we identified differentially expressed genes (DEGs) in clinical melanoma samples. To do so, we considered the median expression of the target molecules of Ibrutinib *BTK*, *EGFR*, *ERBB2*, *JAK3*, *ITK*, and *TEC.* Volcano plots were constructed to visualize the most DEGs, considering the expression level of Ibrutinib target genes (Figure 5A–F).

**Figure 6:**
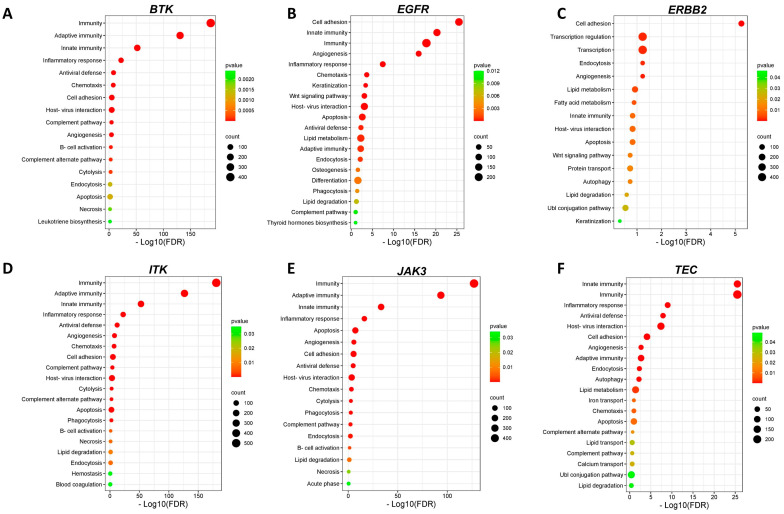
Pathway enrichment analysis. Dot plot of enriched GO biological processes according to −Log10 (FDR) for Ibrutinib targets *BTK* (**A**), *EGFR* (**B**), *ERBB2* (**C**), *ITK* (**D**), *JAK3* (**E**), and *TEC* (**F**). Dot size represents the number of genes in the significant gene list associated with the biological process. Dot color represents the adjusted *p* values.

The functional enrichment analysis performed on DAVID showed that dozens of biological pathways were enriched in melanoma samples according to the expression of Ibrutinib target genes (*p* < 0.05). Among the identified biological processes, necrosis, and apoptosis pathways were enriched considering the DEGs according to the expression of *BTK*, *EGFR*, *ERBB2*, *ITK*, *JAK3*, and *TEC* (Figure 6A–F).

## 4. Discussion

Ibrutinib is a drug that was initially developed as a treatment option for B-cell malignancies. It acts by inhibiting BTK—a protein that plays an essential role in BCR signaling, including BCR-mediated cellular proliferation and survival. It has been described that BTK not only plays important roles in hematopoietic cells but also mediates relevant processes in the tumor microenvironment [20], rendering BTK an interesting and actionable target for treating solid tumors [14]. Importantly, Ibrutinib is not completely specific for BTK, and it has been shown that it modulates more than ten other kinases, which are also inhibited by this drug, including some target proteins commonly associated with solid tumors [14,21]. Indeed, the use of Ibrutinib on solid tumors is currently under investigation for different types of tumors, including melanoma.

In this study, we determined the sensitivity of different melanoma samples to Ibrutinib and showed that Ibrutinib induces cell death in three melanoma cell lines (MeWo, WM164, and SK-MEL-28). Using the MTT assay, we determined that there is a positive dose–response relationship in the cell viability reduction promoted by the drug. Nevertheless, in the following experiments, we only explored the death mechanism events using the IC50 concentration of Ibrutinib, limiting the assessment of functional mechanisms related to other drug doses. 

The MTT assay was followed by the analysis of LDH release by MeWo cells treated with the BTK inhibitor. Both assays revealed that this drug compromises cell viability and influences the cell membrane permeability and integrity [22]. Considering the higher sensitivity of MeWo cells to Ibrutinib as determined by the MTT assay, we continued our investigation using these cells.

Using flow cytometry to detect annexin V and PI staining, as well as caspase 3/7 activity, we identified that apoptosis is an important mechanism involved in the observed cell death events. Interestingly, it was previously demonstrated that Ibrutinib is also capable of inducing autophagy in skin cancer cells [23]. In agreement with our findings, Tan and colleagues showed that exposure to Ibrutinib induces apoptosis in pancreatic cancer cell lines [24]. Another study using a model of liver cancer demonstrated that Ibrutinib could induce apoptosis in SMMC-7721 cells in a dose-dependent manner. Apoptosis related to mitochondrial activity is initiated by the release of cytochrome C and recruitment of caspase 9, which activates effector caspases [25]. It has been demonstrated that Ibrutinib induces breast cancer cell death through apoptosis (caspase-dependent), culminating in the modulation of caspase 3, caspase 8, and PARP1 [26]. Similarly, our results show that Ibrutinib activates caspases 3 and 7 in the MeWo cell line.

In addition, our data demonstrated that the cell death promoted by Ibrutinib was supported by a loss of mitochondrial membrane potential, which is associated with initial apoptotic events [27]. It is worth highlighting that alterations in BAX and BAK are associated with mitochondrial outer membrane permeabilization (MOMP) that leads to apoptosis [28]. In line with this observation, the treatment with Ibrutinib promoted an increased expression of *BAX*, *BAK*, and several other proapoptotic factors in the MeWo cell line, including *ATM*, *HRK*, and *CASP8*.

The induction of cellular apoptosis by Ibrutinib seems to involve different targets. The phosphoinositide 3-kinase (PI3K)/Akt pathway is a pivotal pathway affected by the drug, which acts downstream of BTK and plays a central role in regulating cell survival [29]. Ibrutinib-mediated BTK inhibition also interferes with the nuclear factor–kappa B (NF-κB) pathway, a master regulator of genes involved in apoptosis inhibition and inflammation [30]. Consequently, the downregulation of these survival signals—and possibly others—combined with the disruption of pro-survival pathways ultimately tips the balance in favor of apoptosis, the programmed cell death process.

Considering that protein tyrosine kinases can induce phosphorylation of different target proteins and modulate the activity of their substrates, we hypothesized that, in addition to causing toxicity towards melanoma cells, Ibrutinib would also exert additional anticancer effects. For instance, mitogen-activated protein kinase (MAPK)—a highly relevant mediator in cell proliferation signaling—has also been characterized as a downstream target of Ibrutinib [31]. Furthermore, it has been demonstrated that Ibrutinib can reverse the resistance that some melanoma cells acquire to BRAF inhibitors [32]. Given the importance of cellular proliferation in cancer initiation, progression, and metastasis, as well as the knowledge regarding the role played by Ibrutinib targeting in such processes, we also investigated its effects on cellular division and migratory capacity. Interestingly, our results demonstrated that Ibrutinib exerts a significant antiproliferative effect in the MeWo cell line. Such findings follow prior studies that demonstrated that Ibrutinib inhibits the proliferation of prostate, lymphoma, leukemia, multiple myeloma, pancreatic, and glioblastoma cell lines [24,29,30,33,34,35]. Furthermore, Ibrutinib could inhibit the migratory potential of MeWo cells, indicating that this TKI, in addition to controlling cell proliferation, can also have an anti-metastatic effect. This finding is particularly relevant given the possibility of topical use of Ibrutinib as strategies to prevent melanoma migration.

Recognizing the value of confirming our observations using clinical data, we assessed mRNA data from 144 clinical melanoma samples and investigated whether Ibrutinib target genes would be involved in the cell death and proliferation processes observed in vitro. Therefore, we established DEGs by dichotomizing the Ibrutinib target genes, according to their median expression. We performed an enrichment analysis using the DAVID platform to understand the biological functions associated with the identified DEGs. In line with our functional findings, apoptosis is an enriched pathway identified in the lists of DEGs.

Despite the promising data presented here, recently, a phase II single-arm clinical trial involving 18 patients with metastatic melanoma refractory to PD-1 and MAPK inhibitors did not find an effective antitumor response to the use of Ibrutinib. The authors discuss the reported outcome as a possible consequence of the combination of several factors, including the ability of Ibrutinib to inhibit ITK, the relevance of this target for the treatment of melanoma, and the administered dose (840 mg daily) used to treat solid tumors such as melanoma [36]. Although the result seems disappointing, the authors suggest that additional research is needed to better characterize the Ibrutinib’s mechanisms of action in different types of melanoma cells. One point that raises concern is the high concentration of Ibrutinib required to induce apoptosis in melanoma, as demonstrated in our study. It would be important to investigate the effect of topical use of this TKI on melanoma, but also to explore new therapeutic strategies using Ibrutinib. For example, new approaches such as the use of molecules with bifunctional targeting involving Ibrutinib [37] may also be tested as they show promising tools for the melanoma treatment. Nevertheless, this approach depends on a thorough characterization of the biological effects of each individual compound prior to the design of chimeric molecules. Another interesting approach is the use of nanostructured lipid carriers to enable topical delivery of Ibrutinib in melanoma, as recently demonstrated by our group [38], given the low oral bioavailability that this substance has demonstrated.

## 5. Conclusions

**Figure 7:**
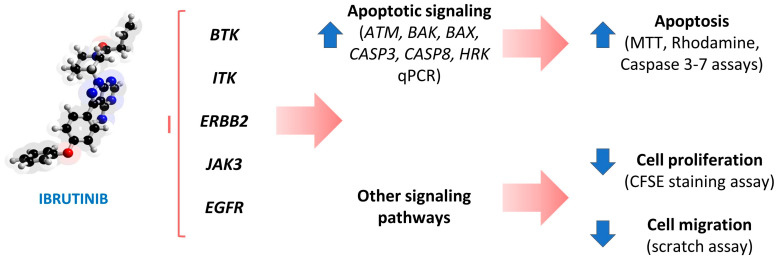
Representative scheme summarizing the anticancer effect of Ibrutinib on MeWo cells. The MeWo cell line presents Ibrutinib target genes *BTK*, *ITK*, *ERBB2*, *JAK3*, and *EGFR*, as confirmed by us through qPCR. After drug treatment, MeWo cells present increased mRNA levels of *ATM*, *BAK*, *BAX*, *CASP3*, *CASP8*, and *HRK*, which are related to apoptotic signaling. Apoptosis then occurs, as shown by MTT viability reduction, as well as by increased mitochondrial membrane permeability (rhodamine assay) and Caspase 3/7 activity. Finally, cell proliferation and cell migration are also compromised by the drug, as shown through the CFSE staining and scratch assays.

Taken together, our results show that Ibrutinib can compromise mitochondrial health by altering their membrane permeability and inducing apoptosis in MeWo cells. Furthermore, we demonstrated that differential expression of all investigated Ibrutinib target genes (i.e., *BTK*, *EGFR*, *ERBB2*, *ITK*, *JAK3*, and *TEC*) is associated with apoptotic mechanisms in clinical melanoma samples (Figure 7). In conclusion, these data show that Ibrutinib can be explored as a therapeutic approach for melanoma, as well as serving as a template for the development of new molecules with anticancer potential.

## Data Availability

The authors confirm that all data underlying these findings are fully available.

## Supplementary Materials

The following supporting information can be downloaded at: https://www.mdpi.com/article/10.3390/biomedicines12051012/s1, Table S1: Primer sequences used in real-time PCR; Table S2: Top 20 upregulated genes in low vs. high *BTK* expression; Table S3: Top 20 downregulated genes in low vs. high *BTK* expression; Table S4: Top 20 upregulated genes in low vs. high *EGFR* expression; Table S5: Top 20 downregulated genes in low vs. high *EGFR* expression; Table S6: Top 20 upregulated genes in low vs. high *ERBB2* expression; Table S7: Top 20 downregulated genes in low vs. high *ERBB2* expression; Table S8: Top 20 upregulated genes in low vs. high *ITK* expression; Table S9: Top 20 downregulated genes in low vs. high *ITK* expression; Table S10: Top 20 upregulated genes in low vs. high *JAK3* expression; Table S11: Top 20 downregulated genes in low vs. high *JAK3* expression; Table S12: Top 20 upregulated genes in low vs. high *TEC* expression; Table S13: Top 20 downregulated genes in low vs. high *TEC* expression; Figure S1: Apoptosis detection by annexin V/PI staining method in SK-MEL-28 cells untreated and treated with IC50 dose of Ibrutinib.
