# Supplementary material for: Ibrutinib Modulates Proliferation, Migration, Mitochondrial Homeostasis, and Apoptosis in Melanoma Cells
Source: Biomedicines. 2024 May 4;12(5):1012. doi: 10.3390/biomedicines12051012 (PMC11117653; doi:10.3390/biomedicines12051012)
Supplement: Supplementary file 1 [file biomedicines-12-01012-s001.zip › biomedicines-2978029-supplementary.pdf]

Supplementary Materials

Supplementary Table S1. Primer sequences used in real-time PCR.

Gene	Primer	Sequence (5'- 3')
GAPDH	Foward	TCAACGACCACTTTGTCAAGCTCAGCT
	Reverse	GGTGGTCCAGGGGTCTTAC
ATM	Foward	GCACAGAAGTGCCTCCAATTC
	Reverse	ACATTCTGGCACGCTTTGG
BAX	Foward	CAGACCGTGACCATCTTTGT
	Reverse	GCCTCAGCCCATCTTCTTC
BAK	Foward	GTTTTCCGCAGCTACGTTTTT
	Reverse	GCAGAGGTAAGGTGACCATCTC
BTK	Foward	TCTGAAGCGATCCCAACAGAA
	Reverse	TGCACGGTCAAGAGAAACAGG
CASP3	Foward	CTAGCGGATGGGTGCTATTG
	Reverse	GATACACAGCCACAGGTATGAG
CASP8	Foward	GGATGGCCACTGTGAATAACTG
	Reverse	TCGAGGACATCGCTCTCTCA
EGFR	Foward	TTCAGGAGCTGTACGTGCATT
	Reverse	CACAAGCGCTGTGTACCCT
ERBB2	Foward	CGGGGTTCCTTCCCCTAATG
	Reverse	CCGAACATCTGGCTGGTTCA
HRK	Foward	AGGTTGGTGAAAACCCTGTG
	Reverse	GCATTGGGGTGTCTGTTTCT
ITK	Foward	GATCAACTGCCTCCACATTGC
	Reverse	GGGCATCACCTCTTAGCACA
JAK3	Foward	GCCTGGAGTGGCATGAGAA
	Reverse	CCCCGGTAAATCTTGGTGAA

Supplementary Table S2. Top 20 upregulated genes in low vs. high *BTK* expression.

Gene	Log2 Ratio	p-Value	q-Value
BTK	-2.02	8.49E-25	1.77E-20
CD86	-1.45	2.87E-14	2.99E-10
CD84	-1.78	7.33E-14	4.80E-10
DOCK2	-1.66	1.02E-13	4.80E-10
SPI1	-1.49	1.15E-13	4.80E-10
IL10	-1.27	2.52E-13	8.72E-10
SLC7A7	-1.51	5.96E-13	1.50E-09
MS4A4A	-1.52	6.08E-13	1.50E-09
LY75	-1.39	6.47E-13	1.50E-09
CYBB	-1.97	8.38E-13	1.74E-09
C3AR1	-1.34	9.89E-13	1.87E-09
HLA-DMA	-1.81	1.38E-12	2.39E-09
MNDA	-1.53	1.49E-12	2.39E-09
CTSS	-1.79	1.66E-12	2.41E-09
CD180	-1.34	1.74E-12	2.41E-09
MPEG1	-1.47	1.85E-12	2.41E-09
TLR7	-1.2	3.22E-12	3.95E-09
HLA-DMB	-1.66	4.29E-12	4.76E-09
CR1	-1.59	4.35E-12	4.76E-09
TFEC	-1.5	4.75E-12	4.94E-09

Supplementary Table S3. Top 20 downregulated genes in low vs. high *BTK* expression.

Gene	Log2 Ratio	p-Value	q-Value
SLC37A3	0.62	5.67E-05	1.65E-03
DBNDD1	0.76	6.27E-05	1.79E-03
KPTN	0.58	1.46E-04	3.53E-03
AHCY	0.55	3.85E-04	7.74E-03
PRR7	0.53	4.28E-04	8.36E-03
MAP1LC3A	0.77	4.44E-04	8.62E-03
LINC00473	0.84	7.82E-04	0.0135
ZBED3	0.18	8.03E-04	0.0138
CBX3	0.42	1.17E-03	0.0184
NT5M	0.46	1.19E-03	0.0186
PDXP	0.41	1.21E-03	0.019
LOH12CR2	0.12	1.30E-03	0.0201
GP1BB	0.67	1.31E-03	0.0201
GAPDH	0.45	1.32E-03	0.0202
VPS37D	0.28	1.39E-03	0.021
DLGAP1	0.88	1.40E-03	0.0211
PROSER2	0.51	1.43E-03	0.0214
LRATD2	0.32	1.75E-03	0.0251
VLDLR	0.73	1.80E-03	0.0257
BUD23	0.46	1.83E-03	0.026

Supplementary Table S4. Top 20 upregulated genes in low vs. high *EGFR* expression.

Gene	Log2 Ratio	p-Value	q-Value
EGFR	-2.54	3.05E-29	6.34E-25
LAMA3	-2.04	3.55E-15	3.70E-11
MPP7	-1.75	2.12E-14	1.20E-10
EPHB6	-2.05	2.31E-14	1.20E-10
PLAC9	-1.66	5.63E-14	2.34E-10
CGNL1	-1.53	6.82E-14	2.36E-10
MFAP4	-1.83	2.43E-13	7.21E-10
FGD5	-1.35	2.97E-13	7.73E-10
CLU	-2.63	9.02E-13	2.09E-09
LAMA2	-1.84	1.34E-12	2.80E-09
SPNS2	-1.04	2.00E-12	3.78E-09
CXCL14	-2.27	2.22E-12	3.84E-09
TEK	-1.11	3.49E-12	5.36E-09
CLEC3B	-2.13	3.61E-12	5.36E-09
TNXB	-2.34	4.21E-12	5.84E-09
SH3RF2	-1.95	4.54E-12	5.91E-09
ACKR1	-1.53	5.16E-12	6.32E-09
TPSAB1	-2.26	6.10E-12	7.05E-09
SCARA3	-1.52	7.29E-12	7.99E-09
ELN	-2.33	8.91E-12	9.27E-09

Supplementary Table S5. Top 20 downregulated genes in low vs. high *EGFR* expression.

Gene	Log2 Ratio	p-Value	q-Value
RABGGTB	0.41	5.65E-04	6.84E-03
PKLR	0.62	8.65E-04	9.49E-03
LIN28B	0.32	1.28E-03	0.0128
HSD3BP5	0.63	1.33E-03	0.0132
NPM1	0.44	1.43E-03	0.0139
TBCE	0.39	1.60E-03	0.0152
TNNI3	0.5	1.74E-03	0.0162
CBX3	0.41	1.78E-03	0.0165
TOMM20P4	0.32	1.79E-03	0.0165
RIOX1	0.72	1.85E-03	0.0169
WNK4	1.15	1.85E-03	0.0169
ERVV-1	0.61	2.20E-03	0.0193
NPM1P24	0.4	2.28E-03	0.0199
ST13	0.36	2.29E-03	0.0199
ZNF695	0.5	2.30E-03	0.02
HSP90AB3P	0.37	2.38E-03	0.0204
LDHBP2	0.09	2.45E-03	0.0209
MTFP1	0.62	2.49E-03	0.0211
MTATP6P1	0.63	3.18E-03	0.0253
CPN1	1.03	3.84E-03	0.0292

Supplementary Table S6. Top 20 upregulated genes in low vs. high *ERBB2* expression.

Gene	Log2 Ratio	p-Value	q-Value
ERBB2	-1.69	2.53E-20	5.25E-16
MRI1	-0.8	3.07E-09	3.19E-05
ZSWIM4	-0.76	7.03E-09	3.35E-05
IMPA2	-1.23	7.25E-09	3.35E-05
PPM1M	-1.18	8.05E-09	3.35E-05
H6PD	-0.97	1.42E-08	4.75E-05
ZNF446	-0.65	1.64E-08	4.75E-05
FES	-1.12	1.83E-08	4.75E-05
SOWAHC	-0.5	2.52E-08	5.82E-05
HNRNPA0	-0.81	3.18E-08	6.62E-05
JDP2	-0.96	5.07E-08	8.59E-05
RABEP2	-0.87	5.52E-08	8.59E-05
PLCD1	-0.97	5.53E-08	8.59E-05
CASKIN2	-0.67	6.15E-08	8.59E-05
PKD1	-0.9	6.42E-08	8.59E-05
PALD1	-0.9	7.53E-08	8.59E-05
MYO15B	-1.31	7.95E-08	8.59E-05
LTB4R2	-1.01	8.50E-08	8.59E-05
RHOB	-1.07	8.78E-08	8.59E-05
P4HTM	-1.11	9.63E-08	8.59E-05

Supplementary Table S7. Top 20 downregulated genes in low vs. high *ERBB2* expression.

Gene	Log2 Ratio	p-Value	q-Value
TRAM1	0.59	3.97E-05	1.99E-03
UQCRB	0.62	1.02E-04	3.31E-03
FTH1P5	0.45	2.10E-04	5.04E-03
WBP11	0.5	2.72E-04	5.85E-03
RPS24P8	0.76	3.03E-04	6.26E-03
TEX15	0.75	3.32E-04	6.63E-03
FTH1	0.52	3.46E-04	6.82E-03
RPL5P34	0.42	3.64E-04	7.05E-03
RPL35P5	0.56	5.14E-04	8.73E-03
GPR19	0.6	6.57E-04	9.94E-03
FTH1P8	0.67	6.69E-04	0.01
GAPDHP63	0.29	7.03E-04	0.0103
TMEM68	0.67	8.00E-04	0.0111
TMCO1	0.42	8.39E-04	0.0114
CSTB	0.57	1.03E-03	0.0129
NT5C3A	0.51	1.16E-03	0.014
ZCCHC9	0.43	1.27E-03	0.0148
MAGOHB	0.43	1.30E-03	0.015
MMGT1	0.39	1.31E-03	0.0151
SEC11C	0.55	1.34E-03	0.0153

Supplementary Table S8. Top 20 upregulated genes in low vs. high *ITK* expression.

Gene	Log2 Ratio	p-Value	q-Value
ITK	-2.56	2.59E-28	5.38E-24
CD3E	-2.39	1.38E-20	1.43E-16
CD6	-2.05	4.08E-20	2.83E-16
GZMA	-2.21	5.24E-19	2.73E-15
TAGAP	-1.7	1.09E-18	4.52E-15
IL2RG	-2.12	3.22E-18	1.12E-14
CORO1A	-2.1	4.46E-18	1.33E-14
BIN2	-1.74	6.04E-18	1.57E-14
IKZF3	-1.98	7.63E-18	1.76E-14
IKZF1	-1.86	1.44E-17	2.94E-14
CD96	-2.03	1.56E-17	2.94E-14
SPN	-1.71	1.80E-17	3.12E-14
CYTIP	-2.02	2.32E-17	3.72E-14
ITGAL	-2.52	2.62E-17	3.85E-14
WDFY4	-1.83	2.78E-17	3.85E-14
PTPN7	-1.93	4.05E-17	5.27E-14
TRBC2	-2.63	5.81E-17	7.11E-14
CD3D	-2.42	8.26E-17	9.39E-14
CD2	-2.14	8.57E-17	9.39E-14
TESPA1	-1.55	9.33E-17	9.71E-14

Supplementary Table S9. Top 20 downregulated genes in low vs. high *ITK* expression.

Gene	Log2 Ratio	p-Value	q-Value
ALX1	1.09	5.35E-06	1.20E-04
PEG10	1.28	2.57E-05	4.68E-04
SPATA17	0.75	5.22E-05	8.62E-04
CHD1L	0.43	1.51E-04	2.19E-03
NELL1	1.8	1.76E-04	2.48E-03
COLGALT2	0.83	1.92E-04	2.68E-03
SAMD13	0.8	1.94E-04	2.71E-03
FDFT1	0.63	2.43E-04	3.26E-03
HIBCH	0.7	2.78E-04	3.66E-03
TRIM51	1.1	4.14E-04	5.13E-03
TRIM51HP	0.28	5.12E-04	6.10E-03
DMRTA1	0.71	5.91E-04	6.87E-03
ZNF106	0.7	9.28E-04	0.01
BFSP1	0.63	1.10E-03	0.0115
TMEM98	0.83	1.19E-03	0.0123
AGMO	1.11	1.26E-03	0.0128
SDCBP	0.65	1.27E-03	0.0129
SGK3	0.79	1.44E-03	0.0143
B3GALT1	0.59	1.74E-03	0.0167
AP3M2	0.47	1.93E-03	0.0182

Supplementary Table S10. Top 20 upregulated genes in low vs. high *JAK3* expression.

Gene	Log2 Ratio	p-Value	q-Value
JAK3	-1.96	1.27E-28	2.64E-24
LCK	-1.84	2.65E-15	1.83E-11
ACAP1	-1.92	2.95E-15	1.83E-11
SPOCK2	-1.7	4.19E-15	1.83E-11
RASAL3	-1.42	4.39E-15	1.83E-11
PSD4	-1.58	7.32E-15	2.54E-11
CD27	-1.73	4.11E-14	1.05E-10
CCDC88B	-1.45	4.33E-14	1.05E-10
CARD11	-1.23	4.56E-14	1.05E-10
FCHO1	-1.32	6.75E-14	1.35E-10
SH2D1A	-1.68	7.15E-14	1.35E-10
ATP2A3	-1.76	1.34E-13	2.33E-10
CD3E	-1.99	3.85E-13	6.14E-10
GIMAP6	-1.35	4.14E-13	6.14E-10
IL21R	-1.29	4.70E-13	6.14E-10
ABI3	-1.33	4.72E-13	6.14E-10
ARHGAP9	-1.65	6.49E-13	7.95E-10
KIF21B	-1.07	8.07E-13	8.79E-10
CD6	-1.7	8.41E-13	8.79E-10
TBC1D10C	-1.44	8.59E-13	8.79E-10

Supplementary Table S11. Top 20 downregulated genes in low vs. high *JAK3* expression.

Gene	Log2 Ratio	p-Value	q-Value
LINC00518	1.32	2.30E-05	4.71E-04
SLC24A5	1.49	6.39E-05	1.09E-03
SAMD13	0.85	7.41E-05	1.23E-03
ALX1	0.95	8.60E-05	1.39E-03
HIBCH	0.74	1.13E-04	1.72E-03
C2CD6	0.87	1.16E-04	1.76E-03
IGSF11	1.08	3.63E-04	4.34E-03
STK32A	1.17	4.67E-04	5.30E-03
PRKD3	0.7	6.02E-04	6.47E-03
FXR1	0.38	6.12E-04	6.55E-03
TDRD3	0.89	6.26E-04	6.69E-03
FAM161A	0.65	6.38E-04	6.79E-03
IFTAP	0.57	8.20E-04	8.25E-03
TEX30	0.54	8.91E-04	8.77E-03
COLGALT2	0.75	9.13E-04	8.94E-03
B3GALT1	0.61	9.92E-04	9.52E-03
MYEF2	0.68	1.06E-03	9.98E-03
TMEM106B	0.34	1.07E-03	0.0101
MT-ND3	0.93	1.23E-03	0.0111
DYRK3	0.6	1.25E-03	0.0113

Supplementary Table S12. Top 20 upregulated genes in low vs. high *TEC* expression.

Gene	Log2 Ratio	p-Value	q-Value
TEC	-1.32	4.12E-22	8.58E-18
GOLGB1	-0.6	2.34E-09	2.28E-05
CDCP1	-1.38	3.97E-09	2.28E-05
IFIH1	-0.95	4.53E-09	2.28E-05
LACTB	-1.05	5.47E-09	2.28E-05
CASP1	-1.48	9.17E-09	2.77E-05
PARP9	-1.04	9.69E-09	2.77E-05
SLC8B1	-0.92	1.06E-08	2.77E-05
KLF3	-0.7	2.45E-08	5.66E-05
MANBA	-0.83	3.02E-08	6.28E-05
DGKG	-1.05	3.57E-08	6.75E-05
SERPINB8	-1.14	7.50E-08	1.28E-04
CCR1	-0.83	8.45E-08	1.28E-04
PARP14	-1.06	8.79E-08	1.28E-04
B4GALT4	-0.76	9.72E-08	1.28E-04
KLHL5	-0.8	9.81E-08	1.28E-04
SLC41A2	-0.92	1.05E-07	1.29E-04
SQOR	-1.12	1.16E-07	1.34E-04
TRIM14	-0.95	1.31E-07	1.44E-04
PLD1	-1.19	1.40E-07	1.46E-04

Supplementary Table S13. Top 20 downregulated genes in low vs. high *TEC* expression.

Gene	Log2 Ratio	p-Value	q-Value
CCT3	0.59	6.85E-06	1.22E-03
AHCY	0.69	7.43E-06	1.29E-03
MEX3A	0.68	3.28E-05	2.69E-03
HMGN2P21	0.51	4.11E-05	2.98E-03
RPL8	0.65	5.39E-05	3.49E-03
RPS2	0.46	6.85E-05	3.88E-03
OBSCN-AS1	0.55	1.37E-04	5.49E-03
RPS18	0.53	1.57E-04	6.05E-03
NPM1	0.51	1.73E-04	6.31E-03
RPL13	0.44	1.83E-04	6.48E-03
DLL3	1.13	2.05E-04	6.90E-03
SOX8	0.64	2.38E-04	7.56E-03
RPL13A	0.4	2.50E-04	7.75E-03
MSI1	0.87	2.72E-04	8.15E-03
RPLP1	0.47	3.26E-04	9.05E-03
RPL23A	0.46	4.71E-04	0.0113
RPS19	0.48	5.59E-04	0.0125
TBCE	0.42	6.26E-04	0.0134
RPL7A	0.43	6.33E-04	0.0134
RPL12	0.46	6.85E-04	0.014

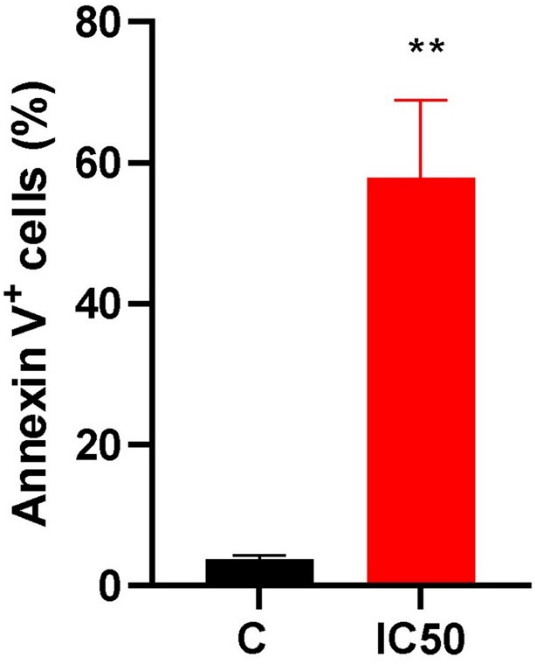

Supplementary Figure S1. Apoptosis detection by annexin V / PI staining method in SK-MEL-28 cells untreated and treated with IC50 dose of Ibrutinib. For flow cytometry analyzes, ten thousand events were recorded for each sample. Data are expressed as mean with SEM and analyzed by Unpaired student t-test. **mean, p<0.01.
